# Clonally related *Escherichia coli* ST69 causing neonatal sepsis in preterm siblings born 1 year apart

**DOI:** 10.1128/asmcr.00221-25

**Published:** 2026-05-29

**Authors:** Dustin D. Flannery, Jie Feng, Alvaro Zevallos Barboza, Robert F. Potter, Karen M. Puopolo, Vivek Mutalik, Scott J. Weissman, Edward G. Dudley

**Affiliations:** 1 Division of Neonatology, Children’s Hospital of Philadelphia, Philadelphia, Pennsylvania, USA; 2 Department of Pediatrics, University of Pennsylvania Perelman School of Medicine 14640, Philadelphia, Pennsylvania, USA; 3 Department of Food Science, The Pennsylvania State University 8082, University Park, Pennsylvania, USA; 4 Department of Pathology & Laboratory Medicine, University of Pennsylvania Perelman School of Medicine 14640, Philadelphia, Pennsylvania, USA; 5 Center for Microbial Medicine, Children’s Hospital of Philadelphia, Philadelphia, Pennsylvania, USA; 6 Lawrence Berkeley National Lab 1666, Berkeley, California, USA; 7 Division of Infectious Diseases, Seattle Children’s Hospital, Seattle, Washington, USA; 8 Penn State E. coli Reference Center, The Pennsylvania State University 8082, University Park, Pennsylvania, USA; Pattern Bioscience, Austin, Texas, USA

**Keywords:** *Escherichia coli*, neonatal sepsis, whole-genome sequencing, vertical transmission, case report

## Abstract

**Background:**

Recurrent neonatal infection caused by the same bacterial lineage across pregnancies is rarely documented. We describe two preterm siblings born one year apart who developed *Escherichia coli* sepsis caused by clonally related strains.

**Case Summary:**

Two female infants were born preterm, 1 year apart, at 26 weeks and 4 days and 30 weeks and 3 days of gestation, respectively, following prolonged premature rupture of membranes. Both infants were evaluated for early-onset sepsis at birth with blood cultures and administered empiric ampicillin and gentamicin; initial blood cultures were sterile, and antibiotics were discontinued after 24 h. At 7 days of age, each infant developed new clinical signs of infection, and both were diagnosed with *E. coli* bacteremia. Both infants recovered after treatment with intravenous antibiotics. Whole-genome sequencing identified the isolates as O15:K52:H18, sequence type 69 (ST69), differing by only a few single nucleotide polymorphisms.

**Conclusion:**

These cases suggest recurrent vertical transmission of a pathogenic *E. coli* lineage leading to neonatal infection arising just beyond the immediate newborn period.

## INTRODUCTION

*Escherichia coli* is the leading cause of both early-onset sepsis (EOS) among preterm infants that is defined as a bloodstream infection or meningitis that occurs within the first 3 days after birth, as well as a major cause of late-onset sepsis (LOS), which occurs after 3 days (1, 2). Despite advances in perinatal care, *E. coli* remains a major contributor to morbidity and mortality among the preterm population. Vertical transmission from colonized mothers is well recognized.

We report two preterm siblings, born 1 year apart, who each developed *E. coli* sepsis at 7 days of age. This unusual presentation raised the question of whether both infections originated from a persistent maternal strain or from unrelated strains circulating in the neonatal intensive care unit (NICU). To address this, we performed whole-genome sequencing to assess strain-relatedness and evaluate the potential role of maternal colonization across pregnancies.

## CASE PRESENTATION

### Sibling 1

A female infant was born at 26 weeks and 4 days of gestation (birth weight 935 g) to a 35-year-old gravida 1, para 1 mother. Pregnancy was complicated by preterm rupture of membranes (PROM) 80 h before delivery, leading to maternal admission to the hospital. On admission, group B *Streptococcus* (GBS) colonization testing was positive. The pregnant patient developed preterm labor and was administered ampicillin, penicillin, amoxicillin, and azithromycin prior to spontaneous vaginal delivery. The infant was admitted to the NICU and evaluated for EOS. Blood was obtained for aerobic and anaerobic cultures, and the infant was empirically treated with ampicillin and gentamicin. The blood cultures were sterile, and antibiotics were discontinued after 24 h. At 7 days of age, the infant was evaluated for LOS due to increased respiratory instability. Blood was obtained from a central venous catheter and peripheral arterial sites; in total, two aerobic and two anaerobic culture bottles were sent, and *E. coli* was isolated from all four cultures. Time to positivity was approximately 8.5 h. By clinical laboratory testing, the isolates were resistant to ampicillin but susceptible to third-generation cephalosporins, aminoglycosides, and carbapenems. Cerebrospinal fluid (CSF) culture was sterile (obtained after initiation of antibiotics), and cell counts were not consistent with meningitis (one red blood cell, four white blood cells, glucose 68, protein 146). Blood cultures obtained at 8 and 9 days of age (after initiation of antibiotics) were sterile. After *E. coli* was identified in blood cultures, the infant was treated with a 10-day course of cefepime (30 mg/kg/dose, intravenous, every 12 h) and ultimately discharged home after a 107-day hospitalization.

### Sibling 2

A female infant was born at 30 weeks and 3 days' gestation (birth weight 1,655 g) to the same now 36-year-old gravida 2, para 2 mother, 1 year after Sibling 1. Pregnancy was complicated by cervical cerclage placement at 12 weeks gestation due to prior preterm labor and subsequent PROM with hospital admission and cerclage removal 82 h before delivery. On admission, the mother tested negative for GBS colonization and developed preterm labor. She was treated with ampicillin and azithromycin prior to vaginal delivery. After spontaneous vaginal delivery, the infant was admitted to the NICU and evaluated for EOS. Blood was obtained for aerobic and anaerobic cultures, and the infant was empirically treated with ampicillin and gentamicin. The blood cultures were sterile, and antibiotics were discontinued after 24 h. At 7 days of age, the infant was evaluated for LOS due to abdominal distention and feeding intolerance. An abdominal radiograph revealed possible intestinal pneumatosis without signs of perforation. Blood was obtained from a peripheral artery, and aerobic and anaerobic culture bottles were sent; *E. coli* was isolated from both cultures. Time to positivity was approximately 11 h. By clinical laboratory testing, the isolates showed the same antibiotic susceptibility as those from Sibling 1. CSF culture was sterile (obtained after initiation of antibiotics), and cell counts were not consistent with meningitis (three red blood cells, one white blood cell, glucose 69, protein 104). Blood cultures obtained at 8 and 9 days of age (after initiation of antibiotics) were sterile. After *E. coli* was identified in blood cultures, the infant was treated with a 10-day course of piperacillin-tazobactam (80 mg/kg/dose [piperacillin component], intravenous, every 6 h ) and ultimately discharged home after a 40-day hospitalization.

*E. coli* isolates and clinical data were collected as part of the Surveillance of Invasive and Resistant *E. coli* in Newborns (SIREN) study, a multicenter repository of neonatal invasive isolates with paired clinical data. The University of Pennsylvania IRB approved the protocol (Protocol #852771) with a waiver of informed consent. Isolates were prepared as frozen glycerol stocks at the hospital microbiology laboratory and shipped to the Penn State *E. coli* Reference Center, where standard biochemical assays and MALDI-TOF mass spectrometry were performed to confirm species. Confirmed isolates were archived; DNA was extracted (Qiagen DNeasy Kit); and sequencing libraries were prepared for whole-genome sequencing on an Illumina MiSeq. Sequences were processed, assembled, and aligned using Trimmomatic (Version 0.39), skesa (Version 2.3.0), and snippy-core (Version 4.6.0), respectively, all available on the GalaxyTrakr public resource (3). Raw reads and assemblies were verified by MicroRunQC (4) to have average raw read Q scores of ≥30, average coverage ≥ 40, *de novo* assembly length of 4.5–5.9 Mbp, and ≤500 contigs. FimH type was determined by FimTyper 1.0 (5). Molecular O antigen typing was performed using SerotypeFinder (6), and molecular K capsule type was assigned using Kaptive (Version 3.0.0b4) with the *E. coli* K antigen database (7, 8). The qualitative match confidence reported by Kaptive was “typable” for all isolates reported here. MUMmer4 was used to identify insertions/deletions between assembled *E. coli* genomes, and CRISPRCasFinder was used to interrogate spacer/repeat composition (9, 10).

Core-genome SNPs were identified by mapping to an ST69 O15:H18 reference genome (BioSample SAMN30184957), which was selected by BLAST of Sibling 1 isolate’s contigs to RefSeq (11, 12). For comparison, we analyzed three other *E. coli* ST69 strains isolated from blood (two) and respiratory (one) cultures obtained from other preterm infants admitted to our NICU during the same period as the two described siblings (2022–2024). Whole-genome sequencing identified all isolates from the two siblings as *E. coli* serotype O15:K52:H18, phylogroup D, sequence type (ST) 69, and *fimH*27. The same eight antimicrobial resistance genes [mdf(A), blaTEM-1b, aph(6)-Id, aph(3″)-Ib, sul2, sul1, dfrA7, and tet(A)] were identified in the three isolates, as were the same four plasmid replicons [IncFIA_1, IncFIB_1, Col440I_1, and IncFII(pCoo)_1]. The two bloodstream isolates from Sibling 1 (designated strains PAH0013-1 and PAH0013-2) differed by two SNPs and by three SNPs compared to the isolate from Sibling 2 (designated PAH0021-1). Screens for insertions and deletions identified a single 122 bp deletion in the Sibling 2 isolate genome compared to the two Sibling 1 isolates, corresponding to loss of two adjacent spacers and repeats within a Type I-E CRISPR-Cas locus (13). In contrast, the three neonatal ST69 isolates from other infants cared for in the NICU during the same period (strains PAH0008-1, PAH0011-1, and PAH0012-1) were separated by 2,217–4,116 SNPs from the sibling isolates, confirming they represented unrelated ST69 lineages in our setting.

## DISCUSSION

We describe neonatal sepsis caused by clonally related *E. coli* ST69 in preterm siblings born 1 year apart, providing genomic evidence of recurrence of the same lineage across pregnancies. ST69 is a lineage commonly associated with extraintestinal infections in adults but less frequently reported in neonatal sepsis (14, 15). Its occurrence in two preterm siblings—each presenting with bloodstream infection at 7 days of age after preterm birth at different gestational ages—highlights both the potential for recurrent vertical transmission of pathogenic species across pregnancies and the importance of strain-level surveillance in neonatal infection.

Our findings have several implications. These sibling cases illustrate the complexity of infection pathogenesis and prevention in preterm infants. EOS typically arises from maternal gastrointestinal or genitourinary colonization, with transmission to the neonate during prolonged rupture of membranes or during labor and delivery. Following acquisition, pathogenic strains may colonize the infant gastrointestinal tract before translocating across immature mucosal barriers to cause bloodstream infection. Studies of EOS and LOS show that microbiology and risk factors shift around 3 days after birth (2, 16). Our cases suggest that fixed-time cutoffs may obscure important aspects of pathogenesis in very preterm infants. Vertical acquisition of GBS, for instance, may not cause infection present at birth but disease may emerge in the days following birth as colonization progresses to bacterial replication and invasion across immature mucosal barriers. Reflecting this, National Healthcare Safety Network standards extend the EOS reporting window for GBS cases up to 6 days of age, recognizing their likely maternal origin (17). This distinction is vital for prevention strategies: measures targeting device-associated, hospital-acquired infections may not prevent vertically acquired infections that arise through mucosal translocation and are not modifiable by standard care practices.

The overlap between EOS and LOS microbiology also informs empiric antibiotic selection. Our cases suggest that antibiotic choice beyond the traditional EOS window should reflect birth circumstances. Prior work showed that preterm infants delivered by cesarean section without PROM or preterm labor differ in EOS risk and maternal microbial exposure from those born after PROM or preterm labor. Shotgun metagenomic sequencing found that by 4–7 days, infants born without PROM or preterm labor were dominated by *Staphylococcus epidermidis*, whereas those born after PROM or preterm labor were dominated by *Enterobacterales* (18). Together with our sibling cases, these findings suggest that consideration should be given to the circumstances of preterm birth when choosing empiric antibiotic therapy for suspected infection, particularly when infection is suspected in the first 1–2 weeks after birth.

Finally, our findings suggest that persistent maternal colonization with pathogenic *E. coli* strains may result in repeated transmission across pregnancies. The most plausible pathway is maternal-to-infant acquisition during rupture of membranes or delivery, followed by colonization of the infant gastrointestinal tract and subsequent translocation into the bloodstream. Whether maternal colonization with pathogenic *E. coli* strains contributes to preterm birth itself—as has been suggested with GBS colonization (19)—requires further investigation. Confirmation in larger studies could enable molecular screening to identify pregnant persons at risk for preterm birth and infants at risk for invasive infection. The absence of maternal isolates is a limitation of our report, precluding definitive source attribution and environmental surveillance cultures from the NICU were not available for comparison. However, the clonal relatedness of isolates across siblings, the rarity of ST69 in the surveillance cohort, and the reproducible clinical presentation make persistent maternal carriage the most plausible explanation.

## Data Availability

The genome data generated in this study are available under NCBI BioSample accessions SAMN50709918 (PAH0021-1), SAMN51096553 (PAH-0013-2), SAMN51096552 (PAH-0013-1), SAMN51096554 (PAH-0012-1), SAMN51096555 (PAH0011-1), and SAMN51096559 (PAH0008-1).
